# Emerging Bacterial Infection: Identification and Clinical Significance of Kocuria Species

**DOI:** 10.7759/cureus.731

**Published:** 2016-08-10

**Authors:** Venkataramana Kandi, Padmavali Palange, Ritu Vaish, Adnan Bashir Bhatti, Vinod Kale, Maheshwar Reddy Kandi, Mohan Rao Bhoomagiri

**Affiliations:** 1 Department of Microbiology, Prathima Institute of Medical Sciences; 2 Medical Director of Clinical Research, Spine Surgery, Tristate Brain and Spine Institute, United States; 3 Microbiology, Chalmeda Anandrao Institute of Medical Sciences

**Keywords:** kocuria spp, urinary tract infection, micrococcaceae, staphylococcus

## Abstract

Recently there have been reports of gram-positive cocci which are morphologically similar to both *Staphylococci* and the *Micrococci*. These bacteria have been identified as *Kocuria* species with the help of automated identification system and other molecular methods including 16S rRNA (ribosomal ribonucleic acid) evaluation. *Kocuria* belongs to the family *Micrococcaceae* which also includes *Staphylococcus* species and *Micrococcus *species. Isolation and clinical significance of these bacteria from human specimens warrant great caution as it does not necessarily confirm infection due to their ubiquitous presence, and as a normal flora of skin and mucous membranes in human and animals. Most clinical microbiology laboratories ignore such bacteria as laboratory and specimen contaminants. With increasing reports of infections associated with these bacteria, it is now important for clinical microbiologists to identify and enumerate the virulence and antibiotic susceptibility patterns of such bacteria and assist clinicians in improving the patient care and management. We review the occurrence and clinical significance of *Kocuria* species*.*

## Introduction and background

*Actinobacteria* are a unique and large group of bacterial species containing five classes, nineteen orders, 50 families and around 220 genera as revealed by the 16S rRNA studies. They have a rigid cell wall and appear Gram-positive or Gram-variable in Gram’s staining. These bacteria are aerobic/facultatively anaerobic and grow better at neutral pH. Many species of *Actinobacteria* are saprophytic and are present in the environment growing under varied conditions as acidophiles, alkaliphiles, halophiles, and thermophiles. Several other actinobacterial species infect plants and animals. Morphologically *Actinobacteria* show different shapes ranging from cocci, coccobacilli, bacilli, and long filamentous bacteria.

*Kocuria* is a Gram-positive cocci arranged in pairs, short chains, tetrads, cubical packets of eight and irregular clusters. *Kocuria* belongs to the phylum *Actinobacteria*, class *Actinobacteria*, order *Actinomycetales*, sub order *Micrococcinae* and family *Micrococcaceae*. This bacterium was first identified and described by Miroslav Kosur, a Slovakian microbiologist. Currently, there are more than 18 species of *Kocuria* identified based on the 16S rRNA phylogenetic studies. The species of *Kocuria* identified thus far include *Kocuria* *assamensis*, *Kocuria* *aegyptia*, *Kocuria* *gwangalliensis*, *Kocuria* *atrinae*, *Kocuria* *carniphila*, *Kocuria* *flava*, *Kocuria* *palustris*, *Kocuria* *halotolerans*, *Kocuria* *himachalensis*, *Kocuria* *koreensis*, *Kocuria kristinae*, *Kocuria* *marina*, *Kocuria* *polaris*, *Kocuria* *rhizophila*, *Kocuria* *rosea*, *Kocuria* *salsicia*, *Kocuria* *sediminis*, *Kocuria* *turfanensis*, and *Kocuria* *varians*. *Kocuria* species (*Kocuria *spp) inhabit the normal skin and mucous membrane of human and animals [1]. *Kocuria* was also isolated from various environmental and ecological niches [2]. These are usually considered as non-pathogenic bacteria which are rarely associated with human infections. Recently there has been a rise in the incidence of infections caused by *Kocuria* spp causing both superficial infections and deep-seated/invasive infections. The cause of concern is that this bacterium appears to have a broad host range involving both immunocompromised as well as immunocompetent individuals. This review attempts to update the morphology, cultural characteristics, pathophysiological properties, and laboratory diagnosis of *Kocuria* spp.

## Review

### Cultural characteristics of Kocuria

**Figure 1:**
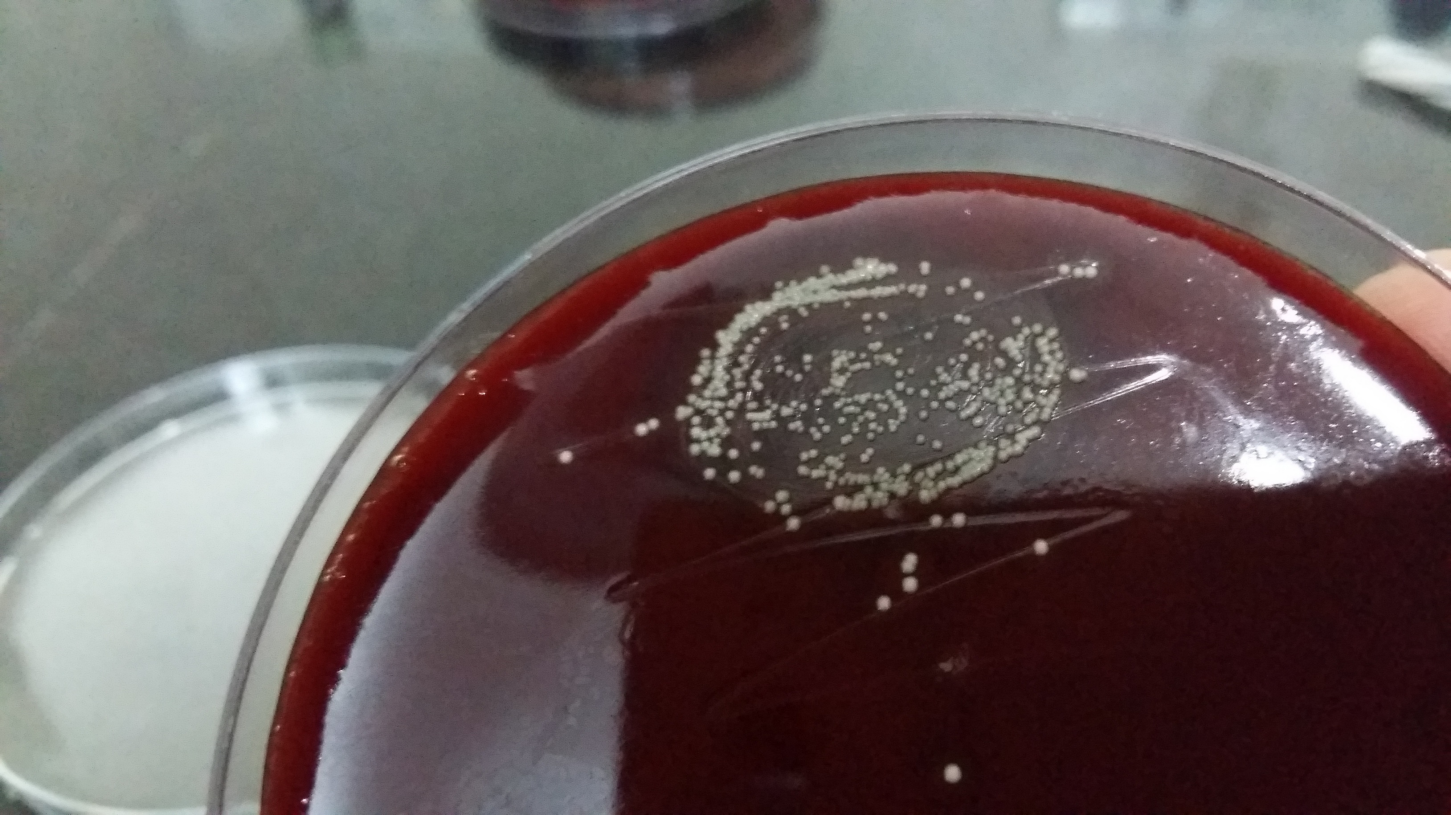
Appearance of Kocuria spp on blood agar after 24 hours of aerobic incubation

*Kocuria* spp do not produce hemolysis on blood agar, unlike most clinical isolates of *Staphylococci. *They usually form 2-3 mm whitish, small, round, raised, convex colonies on initial isolation and might develop non-diffusible yellowish pigmentation after prolonged incubation, as shown in Figure 1.

**Figure 2:**
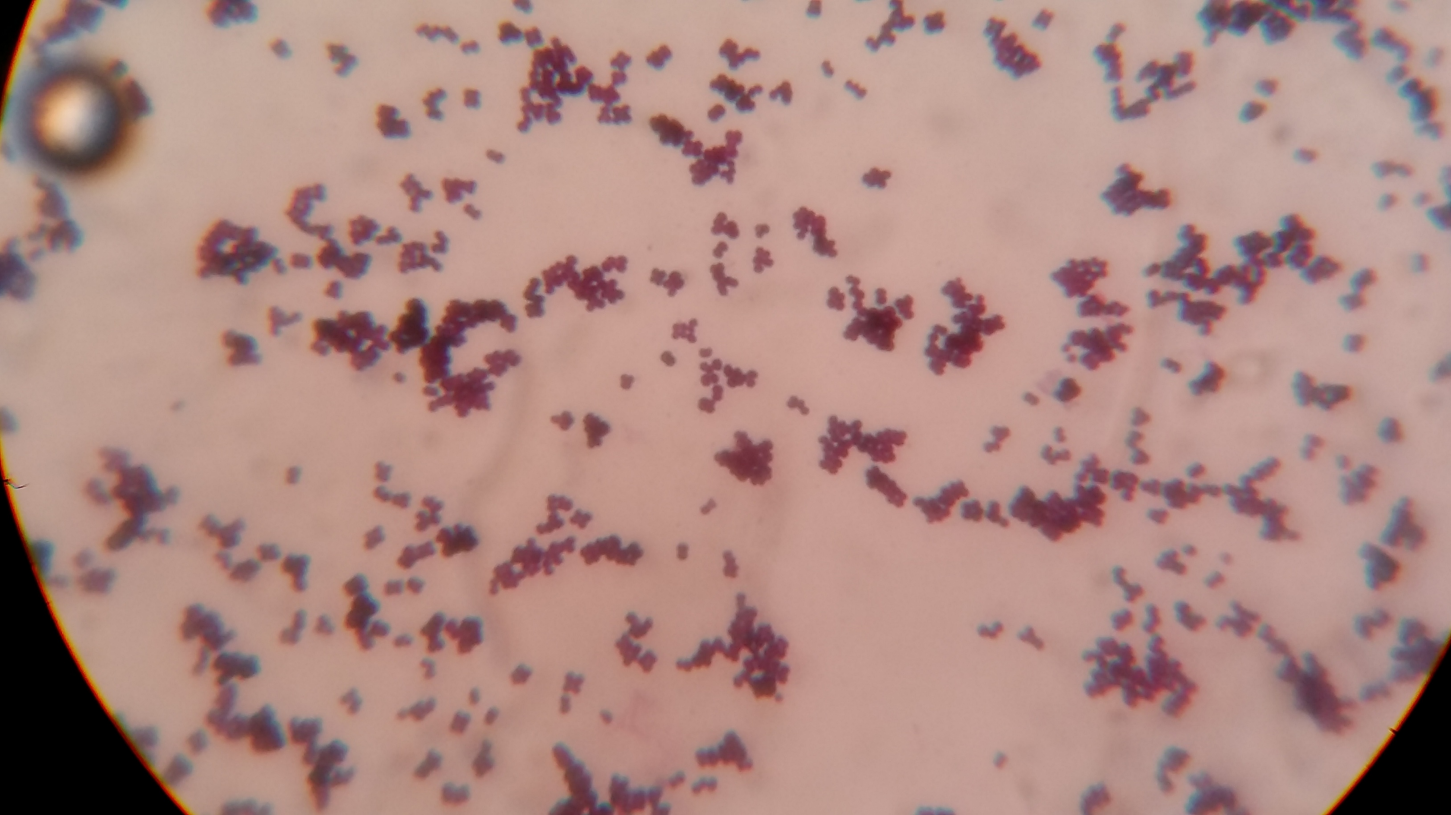
Gram's stain of Kocuria spp showing large sized cocci arranged in pairs, short chains, tetrads, clusters and deeply stained very large cocci

These bacteria appear large and show both tetrads (*Micrococci*) and irregular clusters. An interesting observation in Gram’s stained smear includes the presence of darkly stained and abnormally large clones of cocci, which are not observed in the case of *Staphylococci* and *Micrococci *as shown in Figure 2.

Biochemically these bacteria show great variability by reacting differently towards conventional laboratory identification tests including the catalase, urease, and citrate utilization test. These bacteria are normally negative for mannitol fermentation and coagulase enzyme (both bound and free coagulase).

Susceptibility towards bacitracin and lysozyme and resistance to nitrofurantoin, furazolidone and lysostaphin can be used to separate this bacterium from *Staphylococci*. Modified oxidase test results differentiate between *Kicuria* spp (negative) and *Micrococci*.

### Clinical profile of Kocuria spp

*Kocuria* spp have been reported to be normal flora of human skin and oral cavity and are usually regarded as laboratory contaminants and ignored when isolated in the clinical specimens undermining its pathogenic potential. *Kocuria* was first identified as a causative agent of urinary tract infection way back in 1974, and it was named as *Micrococcus* *kristinae *[3]. Reports of infection with *Kocuria* species have gained prominence in the late twentieth century and are showing an increased trend, signifying its pathogenic potential. Infections associated with isolation of *Kocuria* include urinary tract infections, cholecystitis, catheter-associated bacteremia, dacryocystitis, canaliculitis, keratitis, native valve endocarditis, peritonitis, descending necrotizing mediastinitis, brain abscess and meningitis [4-15]. The predisposing factors associated with infections related to *Kocuria* spp include congenital deformities (short bowel syndrome), chronic catheterization (in cases of total parenteral nutrition), malignancies (ovarian cancer, gastric cancer, myelodysplastic syndrome, acute myelogenous leukaemia, non-Hodgkin’s disease) and patients with end-stage renal disease undergoing continuous ambulatory peritoneal dialysis. Other underlying conditions associated with *Kocuria* infection include diabetes mellitus, tuberculosis, stem cell transplant patients, patients suffering from gallstones, methylmalonic aciduria and pancreatic pseudocyst [16-21].

### Laboratory identification of Kocuria spp

This bacterium is normally misidentified in the clinical microbiology laboratories as coagulase-negative *Staphylococci* (CoNS) based on its gram reaction, catalase positive and coagulase negative properties. Other physiological and biochemical properties of *Kocuria* are the formations of non-hemolytic colonies on blood agar, non-capsulated, non-spore forming, non-motile, non-acid fast and positive for Voges-Proskauer test (VP). It has also been observed that various species of *Kocuria* react differently to routine biochemical tests like the oxidase, amylase, urease, citrate utilization test, gelatinase, phosphatase tests, utilization of inulin, arabinose, N-acetyl-L-glutamic acid, and nitrate reduction tests [22]. This could be attributed to the reason behind the inaccurate identification by both conventional and an automated bacterial identification systems.

The major drawback faced by many laboratories in accurately identifying this bacterium is the need for advanced techniques like 16S rRNA and Matrix-Assisted Laser Desorption/Ionization Time-of-Flight Mass Spectrometry (MALDI-TOF-MS). Although many clinical microbiology laboratories are now equipped with automated identification systems that include VITEK (BioMe´rieux Inc., Durham, NC, USA), VITEK 2 (BioMe´rieux Inc., Durham, NC, USA), API (BioMe´rieux Inc., Durham, NC, USA) and the BD Phoenix™ Automated Microbiology System (BD Diagnostic Systems, Sparks, MD) identification systems, there are studies that have noted false identification of CoNS as *Kocuria* spp and its limitations to identify all the species of *Kocuria *[23]. Previous research has highlighted that in the case of non-availability of molecular and advanced laboratory methods, *Kocuria* can still be identified and differentiated from *Staphylococci* and *Micrococci *using morphological, cultural characteristics and differential antibiotic discs. *Kocuria* spp are sensitive to bacitracin, lysozyme and resistant to nitrofurantoin, furazolidone and lysostaphin [18, 24].

### Antimicrobial susceptibility profile of Kocuria spp

Currently, there are only a few studies that have evaluated the susceptibility profile of *Kocuria* spp. Antimicrobial susceptibility testing results of random case reports are available, although they are still insufficient to establish the exact susceptibility results of *Kocuria* [25]. *Kocuria* spp isolated from a case of peritonitis in a 57 year-old patient suffering from end-stage renal disease revealed sensitivity to ampicillin, cloxacillin, cefotaxime, ciprofloxacin, ofloxacin, levofloxacin, gentamicin, erythromycin, clindamycin, tetracycline, amikacin, linezolid, teicoplanin, vancomycin, imipenem, quinupristin, dalfopristin, rifampicin and was found to be moderately sensitive to ceftazidime [11]. Becker et al. have reported that the *Kocuria* *rhizophila *isolated in blood from a case of sepsis in pediatric age patient revealed resistance only to norfloxacin [21]. Studies by Lee et al., who reported multiple cases involving both extreme age groups noted that *Kocuria* *marina *isolated from peritoneal fluid was resistant only to tetracycline. Other observations by the same authors showed that *K. kristinae *isolated from bacteremia cases revealed resistance to oxacillin, cefazolin and intermediately sensitive to cefotaxime [11]. Resistance to ciprofloxacin and erythromycin was observed in *K. rhizophila *isolated from blood of a 3-year-old catheterized patient as reported by Moissenet et al [26]. *K varians *isolated in peritoneal fluid resistant only to levofloxacin was reported by Meletis et al in a patient undergoing continuous ambulatory peritoneal dialysis (CAPD) [27].

### Recent advances

A recent research report has highlighted the significance of *Kocuria* in causing hospital-acquired infections [13]. The same study has also noted that although *Kocuria* spp are commensals of humans, animals and are present in the environment, they should be considered as potential pathogens in patients who are immunocompromised, undergoing critical care treatment and neonates. A study which included 12 pediatric age patients suffering from underlying debilitating conditions like premature birth and cancer had noted that more than 50% of patients suffered from invasive infections with *Kocuria* spp [28]. Reports of infections caused by *Kocuria* spp among previously healthy and immunocompetent individuals are showing an increased trend. *Kocuria* *rosea *was isolated from a case of descending necrotizing mediastinitis in a 58-year-old woman who was taking medications for gout and hypertension [13]. Another very recent report has observed endocarditis caused by *Kocuria* *rosea *in a 10-year-old female patient. Although the patient was healthy before suffering from the infection, a history of surgery to correct congenital heart disease was present [9]. Evaluation of biofilm production by *Kocuria* spp isolated from a case of peritonitis showed that the strain was negative for biofilm production [18]. Isolation of *K. marina *showing tolerance to severe alkaline conditions in a 7-year-old patient receiving epoprostenol therapy should be considered as an alarming signal regarding the potential of *Kocuria* spp in causing both opportunistic and nosocomial infections [29-30].

## Conclusions

Identification of *Kocuria* spp remains elusive because most clinical microbiology laboratories have limited or no access to advanced molecular techniques. Laboratory identification of *Kocuria* spp can be made conventionally only after high laboratory suspicion. Properties such as morphological variability between these bacteria and other similar gram-positive cocci, as well as biochemical properties including the antimicrobial susceptibility patterns against selective antibiotics could be used to presumptively identify *Kocuria* spp. Infections of *Kocuria *spp normally involve patients with various debilitated conditions. In the era of drug resistance, and prevalence of multi-drug resistant bacteria, occurrence of *Kocuria *spp in hospitalized patients should not always be ignored as contaminants. Further studies emphasizing the determination of the virulence, pathogenic potential, predisposing factors and antimicrobial susceptibility patterns of *Kocuria *spp are warranted.

## References

[1] Stackebrandt E, Koch C, Gvozdiak O (nov). Taxonomic dissection of the genus Micrococcus: Kocuria gen. Cohn.

[2] Park EJ, Kim MS, Roh SW (2010). Kocuria atrinae sp. nov., isolated from traditional Korean fermented seafood. Int J Syst Evol Microbiol.

[3] Tvrzová L, Schumann P, Sedlácek I (2005). Reclassification of strain CCM 132, previously classified as Kocuria varians, as Kocuria carniphila sp. nov. Int J Syst Evol Microbiol.

[4] Mashouf RY, Babalhavaeji H, Yousef J (2009). Urinary tract infections: bacteriology and antibiotic resistance patterns. Indian Pediatr.

[5] Ma ES, Wong CL, Lai KT (2005). Kocuria kristinae infection associated with acute cholecystitis. BMC Infec Dis.

[6] Sohn KM, Baek JY, Kim SH (2015). Catheter-related bacteremia caused by Kocuria salsicia: the first case. J Infect Chemother.

[7] Domont F, Fleche-Mateos AL, Bremond-Gignac D (2014). Kocuria dacryocystitis infection, caused by Kocuria ocularis sp. Nov. JMM Case Rep.

[8] Mattern RM, Ding J (2014). Keratitis with Kocuria palustris and Rothia mucilaginosa in Vitamin A Deficiency. Case Rep Ophthalmol.

[9] Moreira JS, Riccetto AG, Silva MT (2015). Endocarditis by Kocuria rosea in an immunocompetent child. Braz J Infect Dis.

[10] Srinivasa KH, Agrawal N, Agarwal A (2013). Dancing vegetations: Kocuria rosea endocarditis. BMJ Case Rep.

[11] Lee JY, Kim SH, Jeong HS (2009). Two cases of peritonitis caused by Kocuria marina in patients undergoing continuous ambulatory peritoneal dialysis. J Clin Microbiol.

[12] Brändle G, L'Huillier AG, Wagner N (2014). First report of Kocuria marina spontaneous peritonitis in a child. BMC Infect Dis.

[13] Lee MK, Choi SH, Ryu DW (2013). Descending necrotizing Mediastinitis caused by Kocuria rosea: a case report. BMC Infectious Diseases.

[14] Tsai CY, Su SH, Cheng YH (2010). Kocuria varians infection associated with brain abscess: A case report. BMC Infect Dis.

[15] Sipahi OR, Mermer S, Aydemir S (2014). Kocuria rosea meningitis. Surg Infect (Larchmt.

[16] Martinaud C, Gaillard T, Brisou P (2008). Bacteremia caused by Kocuria kristinae in a patient with acute leukaemia. Med Maladies Infect.

[17] Lee M-N, Huh HJ, Kim B (2014). A case of catheter-related Kocuria marina bloodstream infection in a patient with multiple myeloma. Lab Med Online.

[18] Purty S, Saranathan R, Prashanth K (2013). The expanding spectrum of human infections caused by Kocuria species: a case report and literature review. Emerg Microbes Infect.

[19] Citro R, Prota C, Greco L (2013). Kocuria kristinae endocarditis related to diabetic foot infection. J Med Microbiol.

[20] Altuntas F, Yildiz O, Eser B (2004). Catheter-related bacteremia due to Kocuria rosea in a patient undergoing peripheral blood stem cell transplantation. BMC Infect Dis.

[21] Becker K, Rutsch F, Uekötter A (2008). Kocuria rhizophila adds to the emerging spectrum of micrococcal species involved in human infections. J Clin Microbiol.

[22] Savini V, Catavitello C, Masciarelli G (2010). Drug sensitivity and clinical impact of members of the genus Kocuria. J Med Microbiol.

[23] Boudewijns M, Vandeven J, Verhaegen J (2005). Vitek 2 automated identification system and Kocuria kristinae. J Clin Microbiol.

[24] Ben-Ami R, Navon-Venezia S, Schwartz D (2003). Infection of a ventriculoatrial shunt with phenotypically variable Staphylococcus epidermidis masquerading as polymicrobial bacteremia due to various coagulase-negative Staphylococci and Kocuria varians. J Clin Microbiol.

[25] Szczerba I (2003). [Susceptibility to antibiotics of bacteria from genera Micrococcus, Kocuria, Nesterenkonia, Kytococcus and Dermacoccus]. Med Dosw Mikrobiol.

[26] Moissenet D, Becker K, Mérens A (2012). Persistent bloodstream infection with Kocuria rhizophila related to a damaged central catheter. J Clin Microbiol.

[27] Meletis G, Gogou V, Palamouti M (2012). Catheter-related relapsing peritonitis due to Kocuria varians in a patient undergoing continuous ambulatory peritoneal dialysis. Nefrología (Madr.

[28] Chen HM, Chi H, Chiu NC (2015). Kocuria kristinae: a true pathogen in pediatric patients. J Microbiol immunol infect.

[29] Horiuchi A, Kubota N, Hidaka E (2015). Notable alkaline tolerance of Kocuria marina isolate from blood of a pediatric patient with continuous intravenous epoprostenol therapy. J Infect Chemother.

[30] Dotis J, Printza N, Papachristou F (2012). Peritonitis attributable to Kocuria rosea in a pediatric peritoneal dialysis patient. Perit Dial Int.

